# Pre-treatment inflammatory indexes as predictors of survival and cetuximab efficacy in metastatic colorectal cancer patients with wild-type RAS

**DOI:** 10.1038/s41598-017-17130-6

**Published:** 2017-12-07

**Authors:** Jing Yang, Xinli Guo, Manni Wang, Xuelei Ma, Xiaoyang Ye, Panpan Lin

**Affiliations:** 1 State Key Laboratory of Biotherapy and Cancer Center, West China Hospital, Sichuan University, and Collaborative Innovation Center for Biotherapy, Chengdu, China; 2 West China School of Medicine, West China Hospital, Sichuan University, Chengdu, 610041 PR China

## Abstract

This study aims at evaluating the prognostic significance of neutrophil-to-lymphocyte ratio (NLR), platelet-to-lymphocyte ratio (PLR), lymphocyte-to-monocyte ratio (LMR), and systemic immune-inflammation indexes (SII) in metastatic colorectal cancer (mCRC) patients treated with cetuximab. Ninety-five patients receiving cetuximab for mCRC were categorized into the high or low NLR, PLR, LMR, and SII groups based on their median index values. Univariate and multivariate survival analysis were performed to identify the indexes’ correlation with progression-free survival (PFS) and overall survival (OS). In the univariate analysis, ECOG performance status, neutrphil counts, lymphocyte counts, monocyte counts, NLR, PLR, and LDH were associated with survival. Multivariate analysis showed that ECOG performance status of 0 (hazard ratio [HR] 3.608, p < 0.001; HR 5.030, p < 0.001, respectively), high absolute neutrophil counts (HR 2.837, p < 0.001; HR 1.922, p = 0.026, respectively), low lymphocyte counts (HR 0.352, p < 0.001; HR 0.440, p = 0.001, respectively), elevated NLR (HR 3.837, p < 0.001; HR 2.467, p = 0.006) were independent predictors of shorter PFS and OS. In conclusion, pre-treatment inflammatory indexes, especially NLR were potential biomarkers to predict the survival of mCRC patients with cetuximab therapy.

## Introduction

Cetuximab, as a functional antagonist of the EGF and TGF ligand, is a monoclonal antibody that binds to the epidermal growth factor receptor (EGFR), leading to the inhibition of the MAPK pathway and therefore suppresses tumor cell differentiation, proliferation, and angiogenesis to regulates tumor progression^1–5^. In this way, cetuximab has been reported to improve clinical outcomes for patients with wild-type RAS metastatic colorectal cancer (mCRC)^6^. The Combination of cetuximab with chemotherapy is the standard first-line treatment for mCRC patients, especially patients with left-sided mCRC^6,7^. Several studies that focused on the MAPK pathway have identified some potential biomarkers with questionable accuracy, but validated predictors of efficacy to cetuximab are still not available^8–11^.

It has been suggested that systemic inflammatory response plays an important role in the development and progression of cancer, and that several haematological components take part in forming inflammation-based variables associated with survival in various tumor^12–14^. The inflammatory indexes, such as neutrophil-to-lymphocyte ratio (NLR), platelet-to-lymphocyte ratio (PLR), lymphocyte-to-monocyte ratio (LMR) and systemic immune-inflammation index (SII) have been reported to be associated with prognosis in several tumors^15–24^. Moreover, previous studies have reported that inflammation indexes were potential markers predicting survival in mCRC patients, such as patients with synchronous colorectal liver metastasis, patients treated with capecitabine combined therapy, and patients treated with bevacizumab^25–28^.

This study aimed at investigating inflammatory indexes including NLR, PLR, LMR, and SII for their prognostic significance and ability to predict survival in mCRC patients receiving cetuximab. To the best of our knowledge, this is the first study to investigate the role of pre-treatment inflammatory indexes as predictors for prognosis and treatment efficacy of cetuximab in mCRC patients with wild-type RAS.

## Results

### Patient population

A total of 7207 patients with CRC were identified from the database and 95 patients were enrolled in this study. The selection process is shown in the Supplementary Fig. 1. Follow-up time ranges from 12 to 72 months, with the median time of 40 months. At the final follow-up date, 74 (77.9%) of 95 patients had experienced progression of disease, 62 (65.3%) died, and 33 patients (34.7%) were alive. Patients divided into groups on the basis of the median value of each marker, were all comparable for age, gender, ECOG performance status, tumor localization, liver metastasis, lung metastasis, pathological differentiation, M stage and chemotherapy regimen. Baseline characteristics of patients are shown in Table 1. There were 58 males and 37 females with a median age of 56 years (range 27–86). Fifty-five patients (57.9%) had a performance status of 0 while 40 (42.1%) had a performance states of 1. Thirty patients (31.6%) suffered from left colon cancer, 12 (12.6%) surfered from right colon cancer while 53 (55.8%) suffered from rectal cancer. Seventy-one patients (74.7%) with liver metastasis and 24 (25.3%) without, while fourty-three (45.2%) pantients with lung metastases and 52 (54.8%) without. Among those 95 patients, 33 (34.7%), 51 (53.7%) and 11 (11.6%) patients had low, median and high pathological differentiation respectively. Thirty-nine patients (41.0%) were diagnosed at M1a stage while others (59.0%) at M1b. Regarding to the chemotherapy regimen, 26 patients (27.4%) received FOLFOX, and 69 (72.6%) received FOLFIRI (Table 1).

**Table 1:** Baseline characteristics of the study population.

	NLR		*p*	PLR		*p*	LMR		*p*	SII		*p*
	<2.34	≥2.34		<142.00	≥142.00		<4.00	≥4.00		<460.66	≥460.66	
	*n*(%)	*n*(%)		*n*(%)	*n*(%)		n(%)	n(%)		n (%)	n (%)	
Median age, years (range)	58(33–86)	56(27–77)	0.737	62(33–86)	51(35–70)	0.243	50(33–73)	61(33–83)	0.258	58(33–86)	56(27–77)	0.422
Gender												
Male	29(61.7)	29(60.4)	0.898	30(62.5)	28(59.6)	0.770	28(58.3)	30(63.8)	0.583	33(68.8)	25(53.2)	0.144
Female	18(38.3)	19(39.6)		18(37.5)	19(40.4)		20(41.7)	17(36.2)		15(31.3)	22(46.8)	
ECOG performance status												
0	30(63.8)	25(52.1)	0.246	29(60.4)	26(55.3)	0.615	27(56.3)	28(59.6)	0.743	29(60.4)	26(55.3)	0.680
1	17(36.2)	23(47.9)		19(39.6)	21(44.7)		21(43.8)	19(40.4)		19(39.6)	21(44.7)	
Tumor localization												
Left colon	14(29.8)	16(33.3)	0.132	17(35.4)	13(27.7)	0.392	14(29.2)	16(34.0)	478	14(29.2)	16(34.0)	0.098
Right colon	3(6.4)	9(18.8)		4(8.3)	8(17.0)		8(16.7)	4(8.5)		3(6.3)	9(19.1)	
Rectum	30(63.8)	23(47.9)		27(56.3)	26(55.3)		26(54.2)	27(57.4)		31(64.6)	22(46.8)	
Liver metastasis												
Yes	35(74.5)	36(75.0)	0.952	37(77.1)	34(72.3)	0.595	35(72.9)	36(76.6)	0.680	36(75.0)	35(74.5)	1.000
No	12(25.5)	12(25.0)		11(22.9)	13(27.7)		13(27.1)	11(23.4)		12(25.0)	12(25.5)	
Lung metastasis												
Yes	23(48.9)	20(41.7)	0.477	24(50.0)	19(40.4)	0.349	21(43.8)	22(46.8)	0.765	24(50.0)	19(40.4)	0.412
No	24(51.1)	28(58.3)		24(50.0)	28(59.6)		27(56.3)	25(53.2)		24(50.0)	28(59.6)	
Pathological differentiation												
Low	13(27.7)	20(41.7)	0.144	15(31.3)	18(38.3)	0.360	16(33.3)	17(36.2)	0.866	15(31.3)	18(38.3)	0.360
Median	30(63.8)	21(43.8)		29(60.4)	22(46.8)		27(56.3)	24(51.1)		29(60.4)	22(46.8)	
High	4(8.5)	7(14.6)		4(8.3)	7(14.9)		5(10.4)	6(12.8)		4(8.3)	7(14.9)	
M stage												
M1a	17(36.2)	22(45.8)	0.338	18(37.5)	21(44.7)	0.477	21(43.8)	18(38.3)	0.589	17(35.4)	22(46.8)	0.301
M1b	30(63.8)	26(54.2)		30(62.5)	26(55.3)		27(56.3)	29(61.7)		31(64.6)	25(53.2)	
CT regimen												
FOLFOX	15(31.9)	11(22.9)	0.325	12(25.0)	14(29.8)	0.601	13(27.1)	13(27.7)	0.950	15(31.3)	11(23.4)	0.491
FOLFIRI	32(68.1)	37(77.1)		36(75.0)	33(70.2)		35(72.9)	34(72.3)		33(68.8)	36(76.6)	

Abbreviations: NLR, neutrophil-to-lymphocyte ratio; PLR, platelet-to-lymphocyte ratio; LMR, lymphocyte-to-monocyte ratio; SII, systemic immune-inflammation index; ECOG, Eastern Cooperative Oncology Group; n, number.

A p value < 0.05 was considered statistically significant.

### Univariate analysis and Kaplan–Meier curves

**Figure 1:**
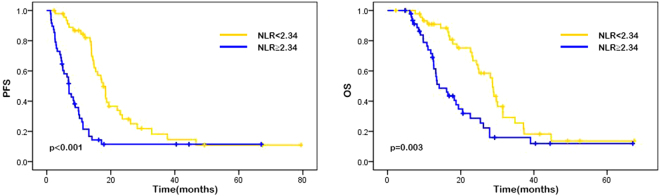
Kaplan–Meier curves of PFS (**a**) and OS (**b**) based on pre-treatment neutrophil-to-lymphocyte ratio (NLR). Elevated NLR was associated with significantly poor PFS (p < 0.001) and OS (p = 0.003).

**Figure 2:**
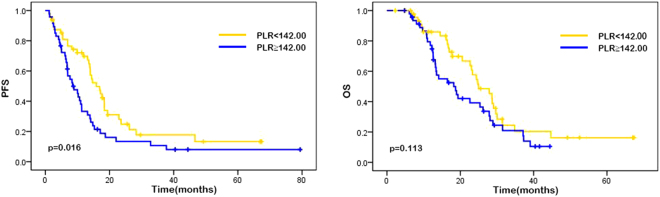
Kaplan–Meier curves of PFS (**a**) and OS (**b**) based on pre-treatment systemic platelet-to-lymphoocyte ratio (PLR). Elevated PLR was significantly associated with poor PFS (p = 0.016) but not with OS (p = 0.113).

**Figure 3:**
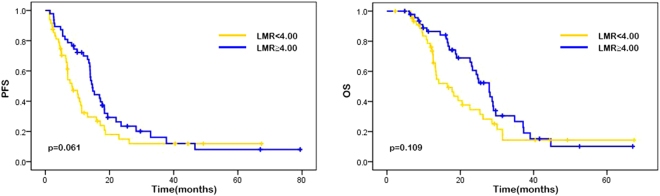
Kaplan–Meier curves of PFS (**a**) and OS (**b**) based on pre-treatment lymphocyte-to-monocyte ratio (LMR). LMR was not significantly associated PFS (p = 0.061) and OS (p = 0.109).

The median progression-free survival (PFS) was 11.00 months (95% CI 11.67–15.57), and the median overall survival (OS) was 17.00 months (95% CI 17.72–23.04). The results of univariate analysis for the association between each variable (gender, age, performance state, tumor localization, liver metastasis, lung metastasis, pathological differentiation, M stage and chemotherapy regimen, neutrophil counts, lymphocyte counts, monocyte counts, platelet counts, NLR, PLR, LMR, SII, lactic dehydrogenase [LDH], carbohydrate antigen 19-9 [CA19-9], and carbohydrate antigen-125 [CA-125]) and PFS or OS are showen in Table 2. In our study, high neutroplil counts, high monocyte counts, high NLR, high PLR and ECOG performance status of 1 were associated with higher risk of disease progression while higher lymphocyte counts was in reverse. As the results suggested that ECOG performance status was the only variable among patient characteristics that significantly affects on survival, patients with performance status of 0 had better median PFS (15.27 vs. 6.40 months, p < 0.001) and OS (25.37 vs. 11.55 months, p < 0.001) than those with performance status of 1. Patients with high neutrophil counts were shown to have significantly worse PFS (14.60 vs. 8.17 months, p < 0.001) and OS (22.97 vs. 13.50 months, p = 0.005), whereas patients with high lymphocyte counts had better PFS (14.07 vs. 6.97 months, p = 0.009) and OS (20.00 vs. 13.40 months, p = 0.037). Patients with high absolute monocyte counts presented shorter median PFS (14.43 vs. 7.92 months, p = 0.018) than patients with low monocyte counts. Patients with high NLR possessed a shorter median PFS (15.90 vs. 6.84 months; p < 0.001) and median OS (24.37 vs. 12.90 months, p = 0.003) compared with those with low NLR. Patients with lower PLR were shown to have a favorable median PFS (13.99 vs. 8.30 months, p = 0.016) compared with those with a higher PLR. Also, patients with high level of LDH were shown to have a poor median PFS (11.60 vs. 9.40 months, p = 0.036). Other factors such as gender, age, tumor localization, liver metastasis, lung metastasis, pathological differentiation, M stage, chemotherapy regimen, platelet counts, LMR, SII, CA19-9, and CA-125 showed no significant associations with survival (Table 2). Similarly, Kaplan-Meier curves showed that performance status of 1 and high NLR were associated with poor PFS (p < 0.001) and OS (p = 0.003), while high PLR was associated with worse PFS (p = 0.016) (Figs 1, 2, 3, Supplementary Figs 2, 3).

**Table 2:** Univariate analysis.

	No.patients	PFS			HR (95%CI)	*p*	OS			HR (95%CI)	*p*
		No. events	Median PFS (months) (95%CI)	*p*			No.events	OS (months) (95%CI)	*p*		
Overall	95	74	11.00 (11.67–17.57)	—	—	—	62	17.00 (17.72–23.04)	—	—	—
Age at diagnosis (years)											
<56	48	37	10.45 (9.38–16.21)		1.00		30	16.07 (14.98–21.89)		1.00	
≥56	47	37	13.17 (11.57–21.40)	0.217	0.834 (0.527–1.320)	0.438	32	19.47 (18.26–26.47)	0.143	0.811 (0.490–1.341)	0.415
Gender											
male	58	45	9.92 (9.91–17.00)		1.00		37	15.67 (15.67–21.75)		1.00	
female	37	29	11.87 (11.12–21.76)	0.330	0.836 (0.522–1.338)	0.456	25	18.70 (18.03–27.97)	0.119	0.806 (0.484–1.341)	0.406
ECOG performance status											
0	55	40	15.27 (15.25–24.33)		1.00		33	25.37 (22.06–29.66)		1.00	
1	40	34	6.40 (5.89–9.13)	<0.001	3.53 (2.125–5.864)	<0.001	29	11.55 (10.91–14.77)	<0.001	5.194 (2.934–9.194)	<0.001
Liver metastasis											
Yes	71	57	10.67 (10.90–16.22)		1.00		44	17.73 (20.01–22.70)		1.00	
No	24	17	11.17 (8.70–26.81)	0.222	0.873 (0.506–1.506)	0.626	18	13.33 (14.16–28.80)	0.635	1.192 (0.687–2.071)	0.532
Lung metastasis											
Yes	43	34	10.00 (9.85–19.68)		1.00		29	16.70 (16.38–23.92)		1.00	
No	52	40	11.32 (10.80–18.20)	0.931	0.929 (0.587–1.470)	0.753	33	17.78 (16.72–24.41)	0.878	0.867 (0.524–1.434)	0.578
M stage											
M1a	39	31	11.00 (10.79–21.13)		1.00		26	19.47 (17.61–27.61)		1.00	
M1b	56	43	10.65 (10.09–17.29)	0.455	1.114 (0.700–1.774)	0.648	36	16.50 (15.88–21.77)	0.166	1.299 (0.738–2.046)	0.428
Chemotherapy regimen											
FOLFOX	26	14	10.99 (9.66–24.64)		1.00		12	12.65 (13.21–24.71)		1.00	
FOLFIRI	69	60	11.00 (10.63–16.71)	0.298	1.553 (0.926–2.603)	0.084	50	18.23 (17.87–23.95)	0.518	1.361 (0.723–2.560)	0.34
Neutrophil counts											
<3.6	46	30	14.60 (13.66–23.42)		1.00		26	22.97 (18.99–27.17)		1.00	
≥3.6	49	44	8.17 (7.67–14.21)	0.010	2.406 (1.497–3.866)	<0.001	36	13.50 (14.41–21.27)	0.050	2.095 (1.255–3.297)	0.005
Lymphocyte counts											
<1.40	47	39	6.97 (7.70–16.04)		1.00		33	13.40 (14.24–20.57)		1.00	
≥1.40	48	35	14.07(13.14–21.50)	0.066	0.542 (0.341–0.860)	0.009	29	20.00 (19.09–27.49)	0.027	0.585 (0.353–0.969)	0.037
Monocyte counts											
<0.37	47	34	14.43 (12.76–22.00)		1.00		31	22.67 (18.50–26.36)		1.00	
≥0.37	48	40	7.92(8.22–15.61)	0.066	1.753 (1.103–2.785)	0.018	31	14.32 (14.73–22.01)	0.130	1.398 (0.843–2.316)	0.194
Platelet counts											
<195	47	35	9.90 (9.74–17.44)		1.00		31	16.50 (15.67–23.45)		1.00	
≥195	48	39	11.15 (11.04–20.21)	0.496	0.995 (0.630–1.572)	0.982	31	18.47 (17.41–24.94)	0.550	0.855 (0.519–1.408)	0.538
NLR											
<2.34	47	34	15.90 (15.22–24.17)		1.00		29	24.37 (20.41–28.12)		1.00	
≥2.34	48	40	6.84 (6.20–13.11)	0.001	2.853 (1.774–4.588)	<0.001	33	12.90 (13.10–20.03)	0.004	2.136 (1.286–3.548)	0.003
PLR											
<142	48	34	13.99 (12.44–21.00)		1.00		28	18.65 (17.78–26.39)		1.00	
≥142	47	40	(8.30–16.61)	0.155	1.769 (1.113–2.810)	0.016	34	13.50 (15.45–21.82)	0.200	1.507 (0.908–2.502)	0.113
LMR											
<4	48	37	6.97 (7.95–15.72)		1.00		31	12.92 (13.84–21.28)		1.00	
≥4	47	37	14.13 (13.02–21.91)	0.058	0.644 (0.406–1.020)	0.061	31	23.10 (19.49–27.01)	0.033	0.664 (0.402–1.095)	0.109
SII											
<460.66	48	36	13.99 (12.42–19.54)		1.00		29	18.40 (17.64–25.10)		1.00	
≥460.66	47	38	7.67 (8.39–18.06)	0.356	1.554 (0.980–2.465)	0.061	33	13.50 (15.45–23.27)	0.456	1.386 (0.841–2.287)	0.201
LDH^a^											
<220	58	41	11.60 (11.82–20.34)		1.00		33	16.92 (16.85–24.27)		1.00	
≥220	36	33	9.40 (8.41–16.03)	0.213	1.636 (1.032–2.594)	0.036	28	18.60 (16.41–24.19)	0.926	1.424 (0.858–2.363)	0.171
CA19-9^b^											
<22	48	39	12.25 (11.73–20.07)		1.00		35	19.62 (17.96–24.63)		1.00	
≥22	42	32	8.62 (8.34–16.35)	0.222	1.249 (0.780–1.998)	0.354	24	15.30 (14.45–22.70)	0.300	1.016 (0.603–1.711)	0.953
CA-125^c^											
<35	40	30	12.62 (10.59–17.66)		1.00		25	17.38 (16.52–24.07)		1.00	
≥35	12	10	7.55 (1.30–34.67)	0.628	1.412 (0.686–2.907)	0.349	7	15.68 (11.08–33.13)	0.679	0.868 (0.375–2.013)	0.742

Abbreviations: ECOG, Eastern Cooperative Oncology Group; CT, chemotherapy; NLR, neutrophil-to-lymphocyte ratio; PLR, platelet-to-lymphocyte ratio; LMR, lymphocyte-to-monocyte ratio; SII, systemic immune-inflammation index; LDH, lactate dehydrogenase, CA19-9, carbohydrate antigen 19-9; CA-125, carbohydrate antigen-125; PFS, progression-free survival; OS, overall survival; HR, hazard ratio; CI, confidence interval.

^a^94 were available; ^b^90 were available; ^c^52 were available.

A p value < 0.05 was considered statistically significant.

### Multivariate analysis

The variables that showed association with PFS or OS in univariate analysis were included in the Cox proportional hazard multivariate models. The results of multivariate analysis for the association between each variable (ECOG performance status, neutrophil counts, lymphocyte counts, monocyte counts, NLR, PLR, and LDH) and PFS or OS are shown in Table 3. The results suggested that neutrophil counts, lymphocyte counts, NLR and ECOG performance status were independent predictors for both PFS and OS. ECOG performance status of 0 was associated with better median PFS (hazard rate [HR] 3.608, 95% confidence interval [CI] 2.096–6.213, p < 0.001) and OS (HR 5.030, 95% CI 2.687–9.417, p < 0.001) than ECOG performance status of 1. High level of neutrophil counts was correlated with unfavorable PFS (HR 2.837, 95% CI 1.664–4.836, p < 0.001) and OS (HR 1.922, 95% CI 1.082–3.414, p = 0.026). High level of absolute lymphocyte counts was correlated with better PFS (HR 0.352, 95% CI 0.210–0.592, p < 0.001) and OS (HR 0.440, 95% CI 0.231–0.692, p = 0.001). Elevated NLR were associated with poor median PFS (HR 3.837, 95% CI 2.117–6.952, p < 0.001) and OS (HR 2.467, 95% CI 1.291–4.717, p = 0.006) (Table 3). In addition, LDH was revealed to be an independent predictive factor of PFS (HR 2.032, 95% CI 1.251–3.300, p = 0.004) but not of OS.

**Table 3:** Multivariate analysis.

	PFS		OS	
	HR(95%CI)	p	HR(95%CI)	p
ECOG performance status (1 vs. 0)	3.608(2.096–6.213)	<0.001	5.030(2.687–9.417)	<0.001
Neutrophil counts (≥3.6 vs. <3.6)	2.837(1.664–4.836)	<0.001	1.922(1.082–3.414)	0.026
Lymphocyte counts (≥1.40 vs. <1.40)	0.352(0.210–0.592)	<0.001	0.440(0.231–0.692)	0.001
Monocyte counts (≥0.37 vs. <0.37)	1.457(0.868–2.445)	0.154	1.029(0.583–1.815)	0.922
NLR (≥2.34 vs. <2.34)	3.837(2.117–6.952)	<0.001	2.467(1.291–4.717)	0.006
PLR (≥142 vs. <142)	1.103(0.632–1.923)	0.731	1.083(0.569–2.060)	0.809
LDH (≥220 vs. <220)	2.032(1.251–3.300)	0.004	1.138(0.660–1.961)	0.643

Abbreviations: NLR, neutrophil-to-lymphocyte ratio; LMR, lymphocyte-to-monocyte ratio; SII, systemic immune-inflammation index; ECOG, Eastern Cooperative Oncology Group; PFS, progression-free survival; OS, overall survival; HR, hazard ratio; CI, confidence interval.

A p value < 0.05 was considered statistically significant.

## Discussion

Increasing evidence suggested that the inflammatory reaction plays an important role in tumor development^29–32^. Accordingly, serum blood cells such as neutrophils, lymphocytes, platelets and monocytes have been assessed in different malignancies and found to be able to predict prognosis and response to treatment^33–35^. Furthermore, several studies have reported that inflammatory indexes including NLR, PLR, LMR and SII were potential prognostic markers for various tumors^36–42^. Such parameters were also associated with survival in mCRC patients, including those receiving bevacizumab or palliative chemotherapy^25,43^. In our study, we observed that pre-treatment inflammatory indexes were potential prognostic factors for survival in mCRC patients receiving cetuximab.

The results of this study suggested that the elevated pre-treatment neutrophil counts, lymphocyte counts and NLR were independent predictors for PFS and OS in mCRC patients receiving cetuximab. PLR, LMR and SII showed no significant association with survival. In addition to inflammatory indexes, we analyzed the associations between patients’ clinical factors (gender, age, ECOG performance status, tumor localization, liver metastasis, lung metastasis, pathological differentiation, M stage, chemotherapy regimen, LDH, CA 19-9, CA-125) and survival. We demonstrated that ECOG performance status was an independent influence factor for both PFS and OS. We also found that patients with low pre-treatment LDH had better PFS but no significant difference in patients’ OS was observed. However, other characteristics such as gender, age, tumor localization, liver metastasis, lung metastasis, pathological differentiation, M stage and chemotherapy regimen, CA 19-9, and CA-125 showed no significant associations with survival.

Neutrophils promote tumor development through facilitating the secretion of circulating growth factors such asinterlukin-1, interlukin-6 and VEGF while lymphocytes play a significant role in anti-tumor response by promoting cytotoxic cell death and inhibiting tumor cell proliferation and migration^14,44–47^. Additionally, neutrophils suppress lymphocytes activities, and therefore suppress the anti-tumor immune response^39^. Tumor-associated macrophages which are derived from circulating monocytes, promote tumor growth, migration, invasion, and metastasis^14,48,49^. Thus, neutrophils and monocytes could promote tumor progression, whereas lymphocytes play an important role in the anti-tumor immunity of the host^14,47^. The role of inflammation in cancer progression is supported by studies which showing that many inflammatory diseases increase the risk of tumors, while aspirin and other non-steroidal anti-inflammatory drugs reduce the risk^14,50–53^. Previous studies suggested that low NLR and high LMR correlated with favorable survival in various cancers, including colorectal cancer, esophagus cancer, gastric cancer and pancreatic cancer^16,36–39,42,54–56^. The results of this study confirmed that pre-treatment NLR was an independent predictor for PFS and OS. A prognostic factor with RR > 2 is considered useful practical value, which indicated that elevated NLR was a powerful predictive indicator of poor outcome^57^. This study indicated that LMR was not significantly associated with survival. However, univariable analysis showed a tendency of improved PFS and OS in patients with high LMR which was not an independent prognostic factor. As a result, further studies are expected to confirm the prognostic value of LMR.

Several studies reported that platelets were related to the angiogenesis and tumor invasion through the increasing production of vascular epidermal growth factor in cancer microenvironment^58,59^. In turn, malignant tumor cells induce platelets aggregation and promote the development of cancer-associated thrombosis^60,61^. As a result, platelets recruited to the tumor microenvironment consequently allow tumor cells to evade immune surveillance and to be protected from physical clearance^61,62^. Thus, cancer progression is not only caused by the intrinsic properties of tumor cells but also stimulated by systemic and local inflammatory reactions. In fact, the role of PLR in the prognosis of CRC patients is still controversial. Several studies supported the positive role of pretreatment PLR as a good marker for CRC patients while several studies did not approve this conclusion^22,27,28,36,42,48,55,63,64^. SII was recently investigated as a prognostic marker in several tumors including esophageal tumor, small cell lung cancer, hepatocellular carcinoma and gastric cancer^65–67^. In the present study, PLR showed significant correlation with PFS but not with OS in univariate analysis. However, no statistically significant correlation was observed about the elevated PLR and poor survival in terms of HR value in the multivariate analysis. Elevated SII indicates high neutrophil counts, high platelet counts and low lymphocyte counts. In this study, we did not confirm the associations of SII with survival. Thus, further studies should be performed to investigate the prognostic value of PLR and SII for the efficacy of cetuximab in mCRC patients.

The limitation of this study lies in its retrospective nature. In addition, our single-center study with a limited number of patients (n = 95) might cause selection bias. Thus, a larger study population, multi-center studies and longer follow-up are needed to validate these results.

In conclusion, this study demonstrated that ECOG performance status, neutrophil counts, lymphocyte counts and NLR were independent predictors for PFS and OS in mCRC patients, while serum level of LDH was independent predictors for PFS but not for OS. Pre-treatment inflammatory indexes, especially NLR were potential biomarkers to predict the survival of mCRC patients with cetuximab therapy, which would hopefully establish a convenient and inexpensive approach to predict of the efficacy of cetuximab in the treatment of metastatic colorectal cancer.

## Materials and Methods

### Patients and inflammatory indexes

We reviewed 7207 colon cancer patients treated at West China hospital between January 2009 and December 2015. Patients who met the following criteria were included: (a) patients with pathological diagnosis of CRC, (b) patients with wild-type RAS mCRC, (c) patients receiving first-line treatment (chemotherapy plus cetuximab), and (d) patients with available and complete basic characteristics, laboratory data and follow-up information. Patients with clinical evidence of acute and chronic inflammation, autoimmune diseases, hematological disorders, or underwent radiotherapy, prior steroid therapy were excluded. The following variables were collected and evaluated in this study: gender, age, ECOG performance status, tumor localization, liver metastasis, lung metastasis, pathological differentiation, M stage and chemotherapy regimen. Laboratory tests results included levels of neutrophil counts, lymphocyte counts, platelet counts, LDH, CA19-9, and CA-125. All of the data were retrieved from electronic patient record system. Laboratory data were obtained within 10 days prior to the initial administration of cetuximab. Blood cell counting was performed with Sysmex hematology analyzers. Patients were staged according to the American Joint Committee on Cancer tumor-node-metastasis (TNM) classification system. NLR and PLR were defined as the absolute counts of neutrophils and platelets respectively, divided by the absolute lymphocyte count. LMR was calculated by dividing the absolute lymphocyte count by the absolute monocyte count. SII was calculated as platelet count × neutrophil count/lymphocyte count.

All patients were followed every month in the first year, every 3 months in the second year and every 6 months thereafter. The start date of follow-up was the date of patients receiving the first dose of cetuximab, and the end of follow-up was December 2016 or death. This study was approved by the Ethics Administration Office of West China Hospital, Sichuan University. An exemption from informed consent in our study was also approved by this Ethics Administration Office. In addition, all methods in this study were performed in accordance with the relevant guidelines and regulations.

### Statistical analysis

Group comparisons on disease-specific variables were performed using Chi-square test for categorical variables and Student’s t test for continuous variables. All p-values were based on two-sided testing, and differences were considered statistically significant when p value is less than 0.05. PFS was defined as the duration from patients primarily receiving cetuximab to the date when radiological evidence of recurrence observed. Patients who died but without progression were not censored to the PFS evaluation. OS was defined as the time interval from patients primary received cetuximab to death from any cause or to the last date of follow-up.

Patients were divided into two groups based on the median index value of NLR (2.34), PLR (142.00), LMR (4.00) and SII (460.66), respectively. NLR ≥2.34, PLR ≥142.00, LMR ≥4.00, and SII ≥460.66 were considered as elevated levels. The cut-off value of neutrophil counts, lymphocyte counts, monocyte counts and platelet counts were their median value, respectively. In the univariate analysis, the log-rank test (at a significance level of 5%) was used to compare the PFS and OS between two groups. Survival curves were estimated using the Kaplan–Meier method. All variables with statistic significance in univariate analysis were further evaluated in the multivariate analysis. We investigated the association of multiple variables with survival using the Cox proportional hazard regression analysis. Estimated hazard ratios (HRs) and their two-sided 95% Confidence Intervals (95% CI) were calculated using the Cox proportional hazard model. All statistical analyses were carried out with SPSS version 22.0 (IBM Corporation, Armonk, NY, USA).

### Ethical approval

This study was approved by the Ethics Administration Office of West China Hospital, Sichuan University.

## Electronic supplementary material

Supplementary material
