# Supplementary material for: Pre-treatment inflammatory indexes as predictors of survival and cetuximab efficacy in metastatic colorectal cancer patients with wild-type RAS
Source: Sci Rep. 2017 Dec 7;7:17166. doi: 10.1038/s41598-017-17130-6 (PMC5719445; doi:10.1038/s41598-017-17130-6)
Supplement: Supplementary file 1 — Supplementary material [file 41598_2017_17130_MOESM1_ESM.pdf]

Pre-treatment inflammatory indexes as predictors of survival and cetuximab efficacy in metastatic colorectal cancer patients with wild-type RAS

Jing Yang, Xinli Guo, Manni Wang, Xuelei Ma, Xiaoyang Ye and Panpan Lin

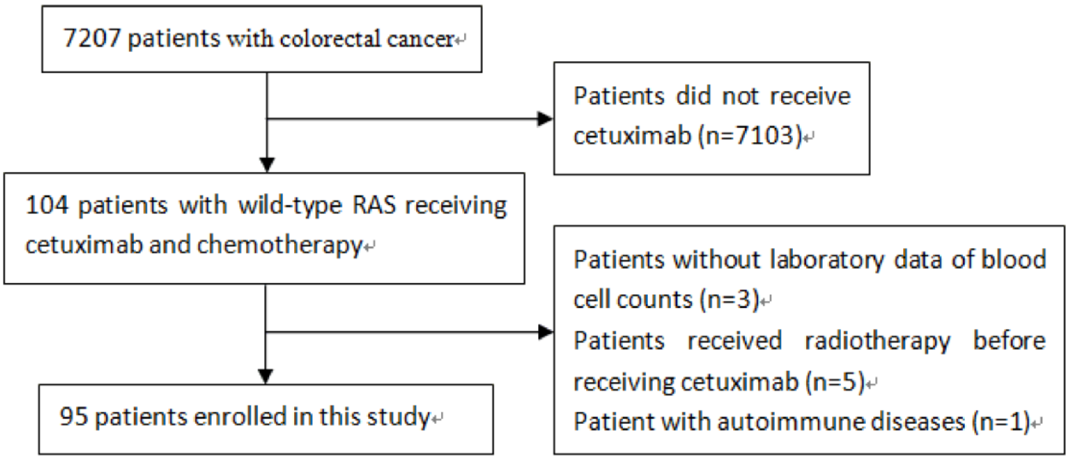

Supplementary figure 1: Selection process of the patients.

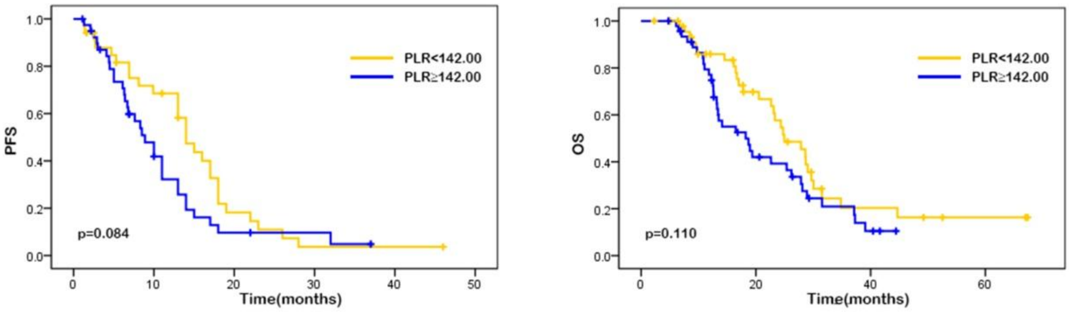

Supplementary figure 2: Kaplan–Meier curves of PFS (a) and OS (b) based on pre-treatment platelet-to-lymphocyte ratio (PLR). PLR was not significantly associated with PFS (p=0.084) and OS (p=0.110).

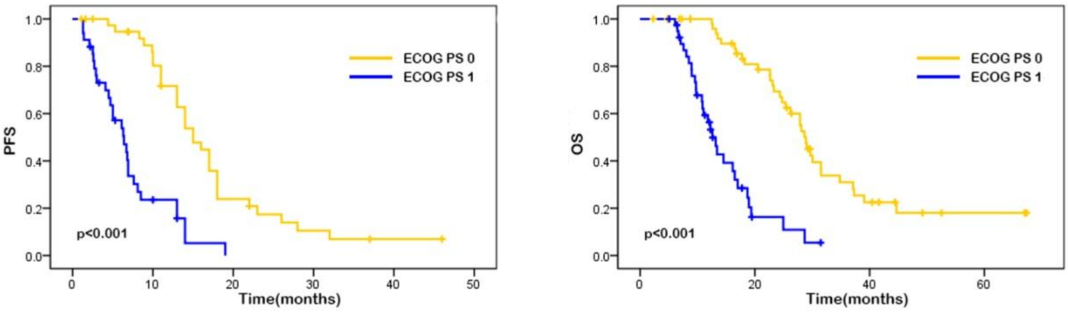

Supplementary figure 3: Kaplan–Meier curves of PFS (a) and OS (b) based on pre-treatment ECOG performance status. ECOG performance status of 0 was associated with significantly better PFS ($p<0.001$) and OS ($p<0.001$).
